# Paul I. Terasaki, Ph.D: a pioneer in transplant medicine and a dedicated philanthropist

**DOI:** 10.3389/frtra.2024.1500493

**Published:** 2024-11-06

**Authors:** Jon Kobashigawa

**Affiliations:** Department of Cardiology, Cedars-Sinai Smidt Heart Institute, Los Angeles, CA, United States

**Keywords:** transplantation, histocompatibility, micro-cytotoxicity, philanthropy, humoral immunity

## Abstract

In the last five decades, remarkable surgical and medical advances ensued within the field of organ transplantation. These strides were marked by significant breakthroughs in transplant immunology, with Dr. Paul I. Terasaki standing as a true pillar of the field. This article highlights major milestones in Dr. Terasaki's life, his groundbreaking accomplishments in the field of transplant medicine, and his enduring philanthropic contributions to numerous medical and community organizations.

In the last five decades, remarkable surgical and medical advances ensued within the field of organ transplantation, supported by the revolutionary achievements of pioneers who helped turn what most considered “experimental” in the 1950s and 1960s into the gold-standard treatment option for patients with end-organ diseases (1). These strides were marked by significant breakthroughs in transplant immunology, with Dr. Paul I. Terasaki standing as a true pillar of the field (2). Dr. Terasaki pushed the boundaries of transplant immunology and histocompatibility science with his transformative contributions to our understanding of tissue typing and humoral rejection (3). In addition, Dr. Terasaki demonstrated an unwavering commitment to humanity through his philanthropic efforts. This commentary highlights major milestones in Dr. Terasaki's life, his groundbreaking accomplishments in the field of transplant medicine, and his enduring philanthropic contributions to numerous medical and community organizations.

Paul I. Terasaki, PhD, was born into a humble family in Los Angeles, California, on September 10th, 1929 (3). In 1942, during World War II, his family was forced to relocate to an internment camp in Arizona, where he lived in one modest room shared with his parents, his two brothers, and his aunt. Dr. Terasaki described their living conditions as poor, and the education he received during those three years of confinement as suboptimal (3). After World War II, his family hesitated to return to California and decided to move to Chicago, where Dr. Terasaki finished high school and then was enrolled as a pre-medicine student at the University of Illinois.

In 1948, feeling it was safe to return to Los Angeles, the Terasakis moved back, and Paul was transferred to the University of California Los Angeles (UCLA) to complete his degree in Zoology. He went on to earn three degrees at UCLA: a bachelor's degree in preventive medicine and public health, as well as a master's and a Ph.D in zoology (4). In 1954, he married Hisako Sumioka, a young artist, and the couple had four children (5).

After graduating, he joined the Department of Surgery at UCLA, where his research initially focused on chicken skin grafts. He developed an interest in transplant tolerance and eventually was awarded a postdoctoral fellowship to work in London for a year under future Nobel Prize laureate Peter Medawar. This experience shaped the rest of his career (3). Dr. Terasaki recalls that at that time, the debate about cellular vs. humoral contribution to graft rejection was ongoing, but he leaned mostly towards B-cells and focused on antibody involvement (3, 6).

Returning to UCLA as an “assistant research zoologist,” Dr.Terasaki worked tirelessly for the next five years, putting in 18-hour days (7, 8). In 1964, he made a major breakthrough by introducing the micro-cytotoxicity test. The test required only 1 microliter—one lambda—of serum to identify human leukocyte antigens (HLA) (Figure 1) (9). The “micro-test” was adopted as a standard in the United States in 1968, and two years later, in 1970, it was adopted as an international standard for genetically matching transplant candidates and recipients. In addition, the test was instrumental in resolving cases of disputed paternity and linking HLA to various diseases (10–12). At that time, Dr. Terasaki linked the presence of preformed antibodies against donor HLA antigens and hyperacute graft rejection (13). This finding set the ground for the need to test for these antibodies and to perform lymphocyte cross-match in patients awaiting transplantation.

**Figure 1:**
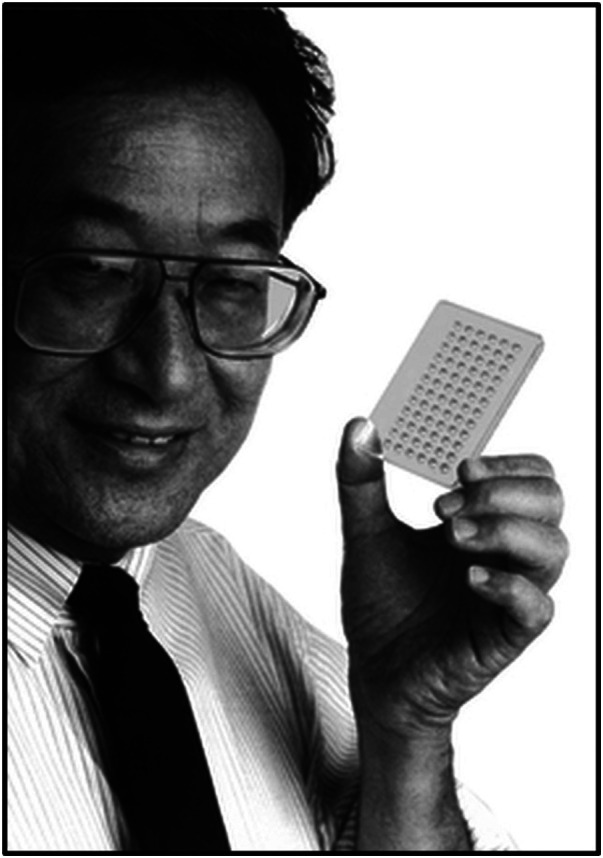
Dr.Paul I. Terasaki holding the Terasaki Plate. Source: https://terasaki.org.

In 1969, Dr. Terasaki became a professor of surgery at UCLA, and he founded the UCLA Tissue Typing Laboratory that year (14). In the 1970s and 80s, the Terasaki lab conducted most of the world's HLA-typing and donor-recipient matching (6). His team developed kits that allowed samples from across the globe to be shipped to Los Angeles with enough numbers of cells for cross-matching (6).

Dr. Terasaki was instrumental in establishing the UCLA kidney registry at UCLA in 1970, which eventually developed into an International Kidney Registry encompassing 52 transplant centers worldwide (15). Dr. Terasaki played a pivotal role in setting the first United Network for Organ Sharing (UNOS) kidney allocation system criteria, and the UCLA kidney transplant he championed served as a precursor for the national transplant database (16).

Additionally, in 1970, he developed, along with Geoffrey Collins, a simple cold storage method to keep donor kidneys viable for longer periods, making it possible to transport organs over long distances (11, 15, 17). In 1984, he founded One Lambda, Inc., a transplant diagnostic company with eight of his former students, to provide diagnostic tools for transplant centers to better match and monitor their patients pre/post-transplant (18).

In 1995, Dr.Terasaki reported that transplants between spouses who were unrelated and poorly HLA-matched had favorable outcomes (19). This led to a significant increase in transplants between spouses, friends, distant relatives, and even complete strangers, expanding the pool of available donors (15). In the late 1990s, One Lambda began to introduce a solid-phase system for identifying HLA antibodies using purified HLA and later recombinant techniques to identify HLA antibodies accurately and laid the groundwork for the creation of the “calculated” percent-reactive antibody, which provides a uniform estimate of how incompatible a transplant candidate is with potential donors (15). It also enabled laboratories to perform a virtual crossmatch, which streamlined organ allocation, significantly increasing the number of transplants among sensitized patients. One Lambda, Inc. was acquired by Thermo Fisher Scientific in 2012 and continues to play a central role in advancing tissue typing (20).

Following Dr.Terasaki's retirement from UCLA in 1999, he founded the Terasaki Foundation, a dedicated research center to study cancer immunotherapy and the role of humoral immunity in organ transplantation (14). Dr. Teraskai's recent contributions to the field transplant have demonstrated that HLA antibodies play a major role in late graft failure, reshaping the transplant community's understanding of chronic graft failure (21).

Dr.Terasaki held numerous leadership positions within the transplant community, including serving as president of the International Transplantation Society and the American Society of Histocompatibility and Immunogenetics, the OPTN/UNOS Board of Directors, and the Histocompatibility and Scientific Advisory Committees (6, 16). Throughout his career, he published over 900 scientific papers, authored more than 20 books, and mentored over 100 postdoctoral scholars (4, 6).

In addition to his groundbreaking scientific achievements, Dr.Terasaki is well-known for his philanthropic endeavors. Throughout his life, he donated $58 million to UCLA to support the Terasaki Life Sciences Building, the UCLA International Institute (later renamed the Paul I. and Hisako Terasaki Center for Japanese Studies), and endowed faculty chairs in that discipline and in U.S.–Japanese relations (4). The Terasakis also established the Nibei Foundation to facilitate fellowship opportunities and partnerships between Japanese and Japanese-American professors and doctors (4).

Paul and his wife, Hisako, were committed to preserving Japanese history in the US. Paul served on many committees, including the Japanese American National Museum in Los Angeles, the Memorial to Japanese American Patriotism in World War II in Washington D.C., and the US-Japan Council, and gave generously to these organizations. After the tsunami, he sponsored 20 Los Angeles college students to volunteer in Japan; the goal was to have the students understand the Tohoku disaster and learn about Japanese history and culture (22). Among many organizations supported by Paul & Hisako Terasaki (Table 1), notable contributions were made to Japanese American Citizens League, Keiro Senior Healthcare, Los Angeles Jewish Symphony, Los Angeles County Museum of Art, Little Tokyo Service Center-(Budukon), Manzanar Committee Smithsonian Institution, The Transplantation Society, the United States-Japan Bridging Foundation, UCLA-The Nikkei Student Union, University of Tokyo, Venice Japanese Community Center, and West LA United Methodist Church. Dr Terasaki also donated an endowed chair in the Department of Surgery at UCLA.

**Table 1:** List of organizations supported by Paul & Hisako Terasaki contributions.

American Association for the Study of Liver Diseases	Lupus Foundation Of Northern California
American Liver Association	Manzanar Committee
American Society for Histocompatibility and Immunogenetics	Mayo Clinic
Asia America Symphony Association	Media Bridges
Cedars Sinai Medical Center	Medical and Health Sciences Foundation
Centenary Sanctuary Fd	Nanka Fukuoka Kenjinkai
Beckman Research Institute—City of Hope	National Inventors Hall of Fame®
Claremont School of Theology	National Japanese American Memorial
DENSHO	Nikkei for Civil Rights and Redress
Foundation FOR Cardiology & Transplant Research	Pacific Lodge Boys Home
Friends of the Smithsonian	Physicians for Social Responsibility
Go For Broke—National Education Center	Pittsburgh Foundation
Grateful Crane Ensemble	Pittsburgh University Medical Center
Harbor-UCLA Hematology	Poston Community Alliance
Higashi Honganji Buddhist Temple	Senior Foundation Charitable Corporation
Japanese American Living Legacy	Smithsonian Institution
Japanese American Cultural & Community Center	The Transplantation Society
Japanese American Citizens League	Toberman Neighborhood Center
Japanese American Memorial to Patriotism During World War II	Tomo No Kai
Japanese American National Museum	Transplant Recipients International Organization
Japanese American Artist Foundation	A Tribute To Issei Pioneers
Japan America Media Association	TRIO
Japan Community Health Care Organization	UCLA-Asian Pacific Alumni
Japanese Language Foundation	Ucla—Japan Tour
JWCH Institute	UCLA Foundation
Kaiser Permanente	UCLA-The Nikkei Student Union
Keiro Senior HealthCare	University Kidney Research Organization
Kizuna	UT Southwestern Medical Center
The Los Angeles Biomedical Research Institute	University Of Tokyo
Los Angeles Jewish Symphony	United States-Japan Bridging Foundation
Los Angeles Philharmonic Association	U.S.-Japan Council
Los Angeles County Museum of Art	Venice Japanese Community Center
Little Tokyo Service Center-(Budukon)	West LA United Methodist Church

Ordered Alphabetically.

In recognition of his achievements, he received numerous prestigious awards, including the Medawar Prize in 1996—the world's highest honor for contributions to the field of transplantation—and the UCLA Medal in 2012, the university's highest distinction (20). In 2013, the American Society of Transplantation honored him as an Innovator in Transplantation (15). For his dedication to preserving the history of Japanese Americans, Terasaki was also awarded the U.S.-Japan Council Lifetime Achievement Award in 2014 (22).

Despite his inauspicious childhood, Dr. Terasaki went on to achieve enormous success as a pioneer in transplant medicine, a mentor to countless health professionals, and a dedicated benefactor to numerous academic, social service, and community organizations. Dr. Terasaki passed away on January 25, 2016, at the age of 86. His legacy will continue to inspire generations to come, and he will be remembered with deep respect and admiration for his groundbreaking contributions and the lives he touched.

## Data Availability

The original contributions presented in the study are included in the article/Supplementary Material, further inquiries can be directed to the corresponding author.
